# Supplementary material for: Self-Reported Non-Celiac Gluten Sensitivity in Brazil: Translation, Cultural Adaptation, and Validation of Italian Questionnaire
Source: Nutrients. 2019 Apr 4;11(4):781. doi: 10.3390/nu11040781 (PMC6521116; doi:10.3390/nu11040781)
Supplement: Supplementary file 1 [file nutrients-11-00781-s001.pdf]

Supplementary Materials. Brazilian non-celiac gluten sensitivity questionnaire

Questionário sobre Sensibilidade ao Glúten Não-Celíaca

1. Idade (anos):
2. Gênero

( ) Masculino

( ) Feminino

3. Grau de Instrução:

( ) Ensino fundamental incompleto

( ) Ensino fundamental completo

( ) Ensino médio incompleto

( ) Ensino médio completo

( ) Ensino superior incompleto

( ) Ensino superior completo

( )Pós graduação incompleta

( )Pós graduação completa

4. Estado civil:
5. Renda mensal familiar:
6. Estado onde mora:

Questões 7–34: Sintomas e Sinais relativos à ingestão de glúten

O glúten é a combinação de dois grupos de proteínas: a gliadina e a glutenina, encontradas dentro de grãos de trigo, cevada e centeio e presente em alimentos como: pão, torrada, bolacha, biscoito, macarrão e outras massas, bolo, cerveja, pizza, salgadinhos, cachorro quente, hambúrguer, gérmen de trigo, triguilho, sêmola de trigo, cereais, barrinha de cereais.

7. Você sente cansaço físico associado à ingestão de glúten?

( ) Sim

( ) Não

8. Você se sente indisposto ou com mal-estar quando ingere glúten?

( )Sim

( )Não

9. Você teve perda de peso associada à ingestão de glúten?

( ) Sim

( ) Não

Quantos quilos perdeu?

___________ Kg (número)

10. Você tem aftas de repetição (feridas na boca) associadas à ingestão de glúten?

( ) Sim

( ) Não

11. Você sente queimação subindo da barriga para o peito ou para a garganta (azia/pirose) quando ingere glúten?

( ) Sim

( ) Não

12. Você sente o ácido subindo da barriga para o peito ou para a garganta (regurgitação ou refluxo ácido) quando ingere glúten?

( ) Sim

( ) Não

13. Você tem dor de estômago associada à ingestão de glúten?

( ) Sim

( ) Não

14. Você tem náuseas ou enjôos associados à ingestão de glúten?

( ) Sim

( ) Não

15. Você tem aerofagia (sensação de engolir muito ar, eventualmente com arrotos) quando ingere glúten?

( ) Sim
( ) Não

16. Você tem distensão abdominal (barriga inchada, estufada) associada à ingestão de glúten?

( ) Sim
( ) Não

17. Você tem dor na barriga associada à ingestão de glúten?

( ) Sim
( ) Não

18. Diarreia

( ) Sim
( ) Não
Número de evacuações por dia: ___________

19. Você tem constipação (intestino preso, fezes endurecidas e dificuldade para evacuar) associada à ingestão de glúten?

( ) Sim
( ) Não

20. Você tem alternância de hábito intestinal (intestino às vezes preso, às vezes solto) associada à ingestão de glúten?

( ) Sim
( ) Não

21. Você tem ou já teve anemia associada à ingestão de glúten?

( ) Sim
( ) Não

22. Você tem dor de cabeça associada à ingestão de glúten?

( ) Sim
( ) Não

23. Você tem dormências associadas à ingestão de glúten?

( ) Sim
( ) Não

24. Você tem sensação de cabeça oca ou raciocínio lento associado à ingestão de glúten?

( ) Sim
( ) Não

25. Você tem dor nos músculos associada à ingestão de glúten?

( ) Sim
( ) Não

26. Você tem dor nas juntas (articulações) associada à ingestão de glúten?

( ) Sim
( ) Não

27. Você se sente desanimado ou deprimido quando ingere glúten?

( ) Sim
( ) Não

28. Você tem ansiedade associada à ingestão de glúten?

( ) Sim
( ) Não

29. Você tem asma associada à ingestão de glúten?

( ) Sim
( ) Não

30. Você tem rinite associada à ingestão de glúten?

( ) Sim
( ) Não

31. Você tem rash cutâneo (lesões na pele como bolhas, manchas, caroços, vermelhidão etc) associado à ingestão de glúten?
( ) Sim
( ) Não
32. Você tem alergias na pele (dermatite) associada à ingestão de glúten?
( ) Sim
( ) Não
33. Você tem algum outro sintoma associado à ingestão de glúten?
Especifique:
34. Qual é a frequência dos sintomas em relação à ingestão do gluten:
( ) Sempre
( ) Frequentemente
( ) Ocasionalmente
35. Quanto tempo depois da ingestão de glúten os sintomas aparecem?
( ) Em 6 horas
( ) Entre 6 e 24 horas
( ) Após 24 horas
36. Os sintomas surgiram quanto tempo antes da hipersensibilidade ao gluten ser detectada?
( ) 1 mês
( ) 6 meses
( ) > 6 meses

Questões 37–42: Distúrbios Associados

37. Você tem algum transtorno alimentar, como por exemplo anorexia (aversão a se alimentar), bulimia (compulsão a ingerir alimentos, seguida de culpa, com vômito ou exercício após), ortorexia (compulsão por ingerir somente alimentos "saudáveis") ou outros?
( ) Sim
( ) Não
( ) Não sei
Qual?
____
38. Você tem Síndrome do Intestino Irritável (dor ou desconforto abdominal recorrente pelo menos 3 dias/mês, nos últimos 3 meses, que melhora com a defecação e/ou se associa com mudança na frequência das evacuações ou com mudança no formato (aparência) das fezes?
( ) sim
( ) não
( ) não sei
39. Você tem alguma outra intolerância alimentar (desconforto digestivo quando ingere corantes, conservantes, lactose, chocolate, vinho...)?
( ) Sim
( ) Não
Qual?
____
40. Você tem alguma alergia?
( ) Sim
( ) Não
Qual?____
41. Você tem alguma doença psiquiátrica (depressão, ansiedade, transtorno bipolar, esquizofrenia...)?
( ) Sim
( ) Não
Qual?____
42. Você tem alguma doença autoimune (como lupus, artrite reumatoide, Sjogren...)?
( ) Sim

( ) Não
( ) não sei
Qual? ____

43. Você tem história de Doença Celíaca na família?
( ) Sim
( ) Não
( ) Não sei
Em quem? ____

44. Quem foi o primeiro a suspeitar que você tinha Sensibilidade ao glúten?
( ) Você mesmo
( ) Algum amigo
( ) O farmacêutico
( ) Um médico (clínico ou de familia)
( ) Um gastroenterogista
( ) Um homeopata
( ) Outro ____

45. Você tem algum teste positivo para doença celíaca?
( ) sim
( ) não
( ) não sei
Se sim, qual?

46. Você tem algum teste positivo para alergia ao trigo?
( ) sim
( )não
( ) não sei
Se sim, qual?
